# Texture Features of ^18^F-Fluorodeoxyglucose Positron Emission Tomography for Predicting Programmed Death-Ligand-1 Levels in Non-Small Cell Lung Cancer

**DOI:** 10.3390/jcm13061625

**Published:** 2024-03-12

**Authors:** Takashi Norikane, Mariko Ishimura, Katsuya Mitamura, Yuka Yamamoto, Hanae Arai-Okuda, Yuri Manabe, Mitsumasa Murao, Riku Morita, Takafumi Obata, Kenichi Tanaka, Makiko Murota, Nobuhiro Kanaji, Yoshihiro Nishiyama

**Affiliations:** 1 Department of Radiology, Faculty of Medicine, Kagawa University, Miki-cho 761-0793, Japan; norikane.takashi.gv@kagawa-u.ac.jp (T.N.); ishimura.mariko@kagawa-u.ac.jp (M.I.); mitamura.katsuya@kagawa-u.ac.jp (K.M.); okuda.hanae@kagawa-u.ac.jp (H.A.-O.); manabe.yuri@kagawa-u.ac.jp (Y.M.); murao.mitsumasa@kagawa-u.ac.jp (M.M.); morita.riku@kagawa-u.ac.jp (R.M.); obata.takafumi@kagawa-u.ac.jp (T.O.); tanaka.kenichi.jt@kagawa-u.ac.jp (K.T.); murota.makiko.kc@kagawa-u.ac.jp (M.M.); nishiyama.yoshihiro@kagawa-u.ac.jp (Y.N.); 2 Department of Internal Medicine, Division of Hematology, Rheumatology and Respiratory Medicine, Faculty of Medicine, Kagawa University, Miki-cho 761-0793, Japan; kanaji.nobuhiro@kagawa-u.ac.jp

**Keywords:** ^18^F-fluorodeoxyglucose, positron emission tomography, programmed death-ligand-1, non-small cell lung cancer, texture

## Abstract

**Background:** Identifying programmed death-ligand-1 (PD-L1) expression is crucial for optimizing treatment strategies involving immune checkpoint inhibitors. However, the role of intratumoral metabolic heterogeneity specifically derived from ^18^F-fluorodeoxyglucose (FDG) positron emission tomography (PET) images in predicting PD-L1 expression in patients with newly diagnosed non-small cell lung cancer (NSCLC) remains unexplored. Here, we investigated the association between FDG PET texture features and PD-L1 expression by retrospectively analyzing the data of patients newly diagnosed with NSCLC who underwent FDG PET/CT scans and PD-L1 immunohistochemical staining before treatment. **Methods:** Patients were categorized based on their tumor proportion scores (TPSs) into negative-, low-, and high-PD-L1 expression groups. We computed the maximum standardized uptake value and 31 texture features for the primary tumor from PET images and compared differences in parameters among the groups. **Results:** Of the 83 patients, 12, 45, and 26 were assigned to the negative-, low-, and high-PD-L1 expression groups, respectively. Six specific texture features (low gray-level run emphasis, short-run low gray-level emphasis, long-run high gray-level emphasis, low gray-level zone emphasis, high gray-level zone emphasis, and short-zone low gray-level emphasis) helped distinguish among all possible combinations. **Conclusions:** Our findings revealed that FDG PET texture features are potential imaging biomarkers for predicting PD-L1 expression in patients newly diagnosed with NSCLC.

## 1. Introduction

The recent emergence of predictive biomarkers has spearheaded the development of novel therapeutic strategies involving targeted therapy and immunotherapy [1]. In patients with non-small cell lung cancer (NSCLC), the expression of programmed death-ligand-1 (PD-L1) on tumor cell surfaces has become a pivotal predictive biomarker guiding treatment decisions with immune checkpoint inhibitors (ICIs) directed against programmed death-1 (PD-1) and PD-L1 [1]. The KEYNOTE-024 trial provided compelling evidence that pembrolizumab outperformed platinum-based chemotherapy in terms of prolonged progression-free and overall survival and fewer adverse events in patients with untreated advanced NSCLC with a PD-L1 tumor proportion score (TPS) of ≥50% [2]. Subsequently, first-line pembrolizumab was also administered to patients with a PD-L1 TPS of ≥1% [3]. Consequently, the assessment of PD-L1 expression is crucial for optimizing ICI treatment strategies. According to the National Comprehensive Cancer Network (NCCN) Clinical Practice Guidelines in Oncology, immunotherapeutic agents can be administered to patients with stage IV NSCLC without actionable mutations. Certain immunotherapeutic agents are recommended as first-line treatment options for patients with PD-L1 expression levels ≥50%. Combination immunotherapy/chemotherapy is also recommended in this setting. Similarly, certain first-line combination immunotherapy/chemotherapy regimens are recommended for patients with PD-L1 expression levels ≥ 1–49%. Patients with PD-L1 levels <1% are prescribed first-line immunotherapy/chemotherapy combinations or chemotherapy alone [4].

While the evaluation of PD-L1 expression levels conventionally relies on specimens obtained through surgical or bronchoscopic procedures, securing satisfactory specimens can pose challenges owing to various factors. Therefore, the development of straightforward and non-invasive surrogates for predicting PD-L1 expression status is of importance. ^18^F-fluorodeoxyglucose (FDG) positron emission tomography (PET) is a highly valuable molecular imaging technique for NSCLC, including its staging, restaging, and prediction of tumor response [5]. Recently, there have been an array of studies exploring the correlation between PD-L1 expression levels and FDG PET parameters, with a primary focus on the maximum standardized uptake value (SUVmax), the most prevalent parameter in clinical practice [6,7,8,9,10]. Nonetheless, these findings have exhibited inconsistency, and a definitive relationship is yet to be established. We recently reported significantly elevated SUVmax in the high-PD-L1 expression group (TPS ≥ 50%) compared with that in the negative-PD-L1 (TPS < 1%) and low-PD-L1 (TPS 1–49%) expression groups. However, no significant distinction in SUVmax was evident between the negative- and low-PD-L1 expression groups [10].

The exploration of tumor heterogeneity assessed through FDG PET images, extending beyond simple measures of tumor radioactivity intensity, such as SUVmax, has gained prominence. Intratumoral metabolic heterogeneity is a critical hallmark of tumor progression, reflecting the molecular complexity of tumor evolution [11]. However, only limited studies have investigated tumor heterogeneity evaluated from FDG PET images as a predictor of PD-L1 expression [12,13,14,15]. Consequently, we aimed to investigate texture features extracted from FDG PET images as potential predictors of PD-L1 expression in patients with newly diagnosed NSCLC. Our findings revealed that FDG PET texture features are potential imaging biomarkers for predicting PD-L1 expression in patients newly diagnosed with NSCLC.

## 2. Materials and Methods

### 2.1. Patients

We conducted a retrospective review of records of patients newly diagnosed with NSCLC who underwent FDG PET/CT scans and PD-L1 immunohistochemical staining of tumor tissue specimens before treatment between August 2014 and January 2021. Patients with incomplete data or inadequate image quality were excluded from the study.

This research adhered to the ethical principles outlined in the 1964 Declaration of Helsinki and its subsequent amendments. This study was approved by the institutional ethical review committee at our institution, and the requirement for obtaining written informed consent was waived owing to the retrospective observational nature of the study.

### 2.2. FDG PET/CT Imaging and Analysis

Most of the methods used in this study followed our previously described methodology [16]. FDG was produced using an automated synthesis system equipped with an HM-18 cyclotron (QUPID; Sumitomo Heavy Industries Ltd., Tokyo, Japan). PET/CT scans were performed with a Biograph mCT 64-slice scanner (Siemens Medical Solutions USA Inc., Knoxville, TN, USA). Patients observed a fasting period of at least 5 h before receiving intravenous FDG injection (5.5 MBq/kg). Normal glucose levels were confirmed before the injection. Emission data were collected after a 90 min rest period, encompassing the area from mid-cranial to proximal thighs (2 min per bed position). Non-contrast low-dose CT scans of the same region were performed for attenuation correction and image fusion. The PET data were reconstructed using a Gaussian filter with an ordered subset expectation maximization algorithm, which included corrections with a point-spread function and a time-of-flight model (2 iterations, 21 subsets).

A board-certified nuclear medicine physician conducted the PET/CT image analysis. The volume of interest (VOI) for the primary tumor was determined using a 40% SUVmax threshold. We assessed the SUVmax and 31 textural features of the primary tumor using the LIFEx package [17]. Texture features were derived from four distinct matrices computed for each VOI, including the gray-level co-occurrence matrix (GLCM), gray-level run length matrix (GLRLM), neighborhood gray-level dependence matrix (NGLDM), and gray-level zone length matrix (GLZLM) (Table S1) [18].

### 2.3. Immunohistochemical Staining for PD-L1

Tissue specimens from primary tumors were obtained through either surgical resection or biopsy. PD-L1 expression was immunohistochemically assessed using PD-L1 22C3 PharmDx (Dako, Carpinteria, CA, USA). The TPS was recorded as the percentage of PD-L1-positive tumor cells relative to all tumor cells. Patients were categorized into negative- (TPS < 1%), low- (TPS 1–49%), and high- (TPS ≥ 50%) PD-L1 expression groups based on their TPS.

### 2.4. Statistical Analysis

IBM SPSS Statistics version 28 (IBM Corp., Armonk, NY, USA) was used for all statistical analyses. Differences in PET parameters among the three groups, categorized by PD-L1 expression levels, were assessed using the Kruskal–Wallis test. Post hoc analysis was carried out using the Bonferroni-corrected Mann–Whitney U test. Statistical significance was set at *p* < 0.05.

## 3. Results

Complete data were obtained for 108 patients; the data of 25 patients were excluded from textural analysis owing to inadequate FDG uptake by the primary tumor. Consequently, 83 patients (53 men and 30 women; mean age, 74 years; age range, 42–92 years) were selected for retrospective analysis. Histologically, 66 patients had adenocarcinoma, 15 had squamous cell carcinoma, and 2 had adenosquamous cell carcinoma.

Tissue specimens for PD-L1 immunohistochemical staining were obtained through surgical resection and biopsy in 22 and 61 patients, respectively. The number of patients in the negative-, low-, and high-PD-L1 expression groups was 12, 45, and 26, respectively. Clinical characteristics according to PD-L1 expression levels are summarized in Table S2.

**Figure 1:**
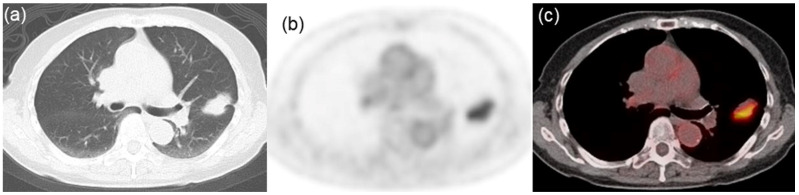
Representative images from an 81-year-old woman with lung adenocarcinoma characterized by negative PD-L1 expression. (**a**) CT image indicating a lung mass in the left upper lobe. (**b**) FDG PET and (**c**) PET/CT fusion images exhibiting high tracer uptake within the tumor (SUVmax = 5.23, LGRE = 0.0107, SRLGE = 0.0099, LRHGE = 158, LGZE = 0.0120, HGZE = 107, and SZLGE = 0.0055).

**Figure 2:**
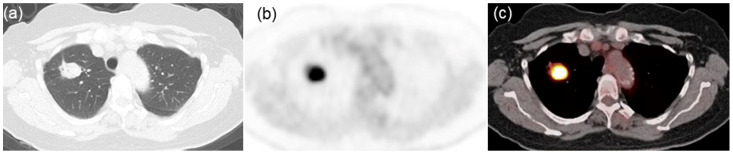
Representative images from a 69-year-old woman with lung adenocarcinoma characterized by low PD-L1 expression. (**a**) CT image displaying a lung mass in the right upper lobe. (**b**) FDG PET and (**c**) PET/CT fusion images exhibiting intense tracer uptake within the tumor (SUVmax = 11.56, LGRE = 0.0021, SRLGE = 0.0020, LRHGE = 685, LGZE = 0.0021, HGZE = 622, and SZLGE = 0.0017).

**Figure 3:**
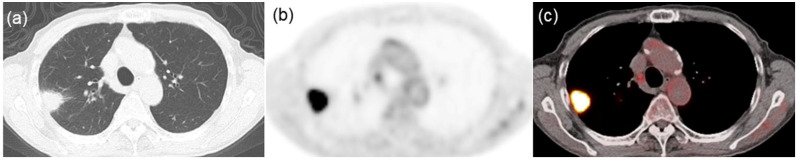
Representative images from an 85-year-old man with lung squamous cell carcinoma characterized by high PD-L1 expression. (**a**) CT image displaying a lung mass in the right upper lobe. (**b**) FDG PET and (**c**) PET/CT fusion images exhibiting intense tracer uptake within the tumor (SUVmax = 21.42, LGRE = 0.0006, SRLGE = 0.0006, LRHGE = 2083, LGZE = 0.0007, HGZE = 1919, and SZLGE = 0.0006).

Descriptive statistics for the three groups, categorized by PD-L1 expression levels, are presented in Table 1. All three groups exhibited a significant difference in SUVmax (*p* < 0.001). Of the 31 assessed texture features, 17 were significantly different among the groups: homogeneity (*p* = 0.034), contrast (*p* = 0.003), entropy (*p* = 0.011), dissimilarity (*p* = 0.004), low gray-level run emphasis (*p* < 0.001), high gray-level run emphasis (*p* < 0.001), short-run low gray-level emphasis (*p* < 0.001), short-run high gray-level emphasis (*p* < 0.001), long-run low gray-level emphasis (*p* < 0.001), long-run high gray-level emphasis (*p* < 0.001), contrast (*p* = 0.034), low gray-level zone emphasis (*p* < 0.001), high gray-level zone emphasis (*p* < 0.001), short-zone low gray-level emphasis (*p* < 0.001), short-zone high gray-level emphasis (*p* < 0.001), long-zone low gray-level emphasis (*p* = 0.013), and zone length non-uniformity (*p* = 0.003). Notably, post hoc comparisons revealed that six texture features—low gray-level run emphasis, short-run low gray-level emphasis, long-run high gray-level emphasis, low gray-level zone emphasis, high gray-level zone emphasis, and short-zone low gray-level emphasis—could distinguish among all combinations of the three groups. Typical radiological images from negative-, low-, and high-PD-L1 expression groups are shown in Figure 1, Figure 2 and Figure 3, respectively.

## 4. Discussion

Considering the therapeutic advantages offered by ICIs in NSCLC, comprehensively assessing PD-L1 expression levels before initiating treatment is crucial. In this study, we investigated FDG PET parameters as predictors of PD-L1 expression in patients with newly diagnosed NSCLC. While SUVmax exhibited a significant difference between the groups, no significant distinction was observed between the negative- and low-PD-L1 expression groups. SUVmax represents the highest FDG uptake within the tumor and reflects a single voxel within a region. In contrast, texture analysis can capture potential spatial variability in tumors, intravoxel intensity heterogeneity, and tracer uptake, providing a more comprehensive tumor characterization. In this study, we identified six texture features that could help distinguish between all combinations among the three groups based on PD-L1 expression levels. Texture features may have the potential to convey more metabolic information about tumor behavior than SUVmax.

Radiomics, initially proposed by Lambin et al., aims to enhance medical image analysis by extracting high-throughput image features not evident through visual interpretation [19]. Pyka et al. demonstrated that textural features from FDG PET could help predict disease-specific survival in patients with early stage NSCLC undergoing primary stereotactic radiotherapy [20]. Some studies have demonstrated the relationship between PD-L1 expression and FDG PET parameters in primary lung cancer; however, the criteria for PD-L1 positivity vary. In our study, we divided patient data into three groups using PD-L1 cut-off values of 1% and 50%. Zhang et al. evaluated the association between FDG PET/CT radiomics and PD-L1 status in 58 patients with NSCLC by dividing them into two groups based on the 1% cut-off for PD-L1 TPS [13]. They observed that lower LGRE and SRLGE were related to PD-L1 positivity [13]. Concordantly, we observed that LGRE and SRLGE decreased with increasing PD-L1 TPS. Conversely, Kim et al. divided 31 patients with NSCLC into two groups based on the median PD-L1 mRNA levels and observed that higher LGRE, SRLGE, and LRLGE were associated with PD-L1 positivity [12]. However, conflicting results exist across different studies, likely because of variations in patient populations, PD-L1 cut-off values, PET instrumentation, and radiomic methodologies. In our study, PD-L1 expression was assessed using immunohistochemistry, whereas Kim et al. investigated PD-L1 mRNA levels. PD-L1 expression, which is typically determined using immunohistochemistry, is confounded by factors such as varying sensitivities to different antibodies and cut-off points of positivity [21]. The texture analysis of PET images can provide insights into biological processes such as glucose metabolism, hypoxia, angiogenesis, and necrosis [22,23]. In our study, six texture features enabled us to distinguish among all combinations of the three groups according to PD-L1 expression (TPS < 1%, 1–49%, and ≥50%). Although some studies have categorized patients into two groups based on PD-L1 levels, distinguishing among these three groups is crucial for optimizing ICI treatment benefits. Phenotypic information derived from radiographic images may be relevant to PD-L1 expression. However, limited studies have evaluated the association between glucose metabolic heterogeneity and PD-L1 expression within tumors, with conflicting results.

Our study has a few limitations. This study is inherently limited by its retrospective design and small number of patients. We did not adjust for important variables or perform statistical association tests such as multivariable or multivariate regression. Therefore, further studies with larger patient cohorts must be conducted to determine whether the significant relationships observed here are truly independent. We examined only PD-L1 expression and did not consider genetic mutations such as epidermal growth factor receptor (EGFR) and Kristen rat sarcoma viral (KRAS) mutations. While a few studies have reported an association between PD-L1 expression and driver mutations, a recent meta-analysis showed that PD-L1 expression is correlated with EGFR mutation but not with anaplastic lymphoma kinase (ALK) rearrangement and KRAS mutations [24]. In contrast to that in NSCLC, PD-L1 expression in other cancers such as head and neck squamous cell carcinoma is scored using the combined positive score (CPS), which encompasses PD-L1 expression on both tumor cells and immune cells in the tumor microenvironment [25]. Ulas et al. reported that CPS differentiated overall survival better than TPS in patients with advanced NSCLC who received ICI monotherapy [26]. However, it is not clear whether CPS is a better predictive biomarker than TPS in NSCLC because of limited availability of related reports. Therefore, further prospective studies with larger patient cohorts are required to validate these preliminary findings and explore the broader application of FDG PET texture features for evaluating PD-L1 expression, which is a crucial factor in deciding the optimal ICI treatment in clinical practice. We intend to expand this research by investigating other cancer types while focusing on various targets.

The role of PD-L1 expression in predicting the efficacy of ICIs has been confirmed by several studies; however, recent evidence indicates that even patients with negative PD-L1 expression many show responses, especially those receiving immunotherapy-based combination therapy [27]. In the present study, the effectiveness of ICIs was not investigated. Recently, Wang et al. reported that dynamic FDG uptake parameters may be useful in distinguishing responders from non-responders, regardless of PD-L1 expression [28]. Therefore, there is an urgent need to develop superior biomarkers of response to immunotherapy or immunotherapy-based combination therapy in patients with NSCLC.

In conclusion, our preliminary findings suggest that six FDG PET texture features may serve as potential non-invasive biomarkers for predicting PD-L1 expression in patients newly diagnosed with NSCLC when tissue samples are not available. Additional studies with larger patient cohorts are needed to validate these findings.

## Figures and Tables

**Table 1:** Descriptive statistics for the three groups categorized by PD-L1 expression levels.

FDG PET Parameter	Negative PD-L1 (*n* = 12)	Low PD-L1 (*n* = 45)	High PD-L1 (*n* = 26)	*p*	*p* for Negative vs. Low PD-L1	*p* for Negative vs. High PD-L1	*p* for Low vs. High PD-L1
SUVmax	8.10 ± 3.65	13.01 ± 7.69	17.71 ± 7.06	**<0.001**	0.050	**<0.001**	**0.045**
Homogeneity	0.40 ± 0.10	0.28 ± 0.12	0.26 ± 0.11	**0.034**	0.269	**0.030**	0.471
Energy	0.016 ± 0.013	0.008 ± 0.029	0.054 ± 0.029	0.059	NA	NA	NA
Contrast	13.9 ± 18.3	41.5 ± 30.3	63.4 ± 48.7	**0.003**	0.072	**0.002**	0.187
Correlation	0.31 ± 0.16	0.40 ± 0.16	0.39 ± 0.13	0.529	NA	NA	NA
Entropy	1.88 ± 0.35	2.18 ± 0.39	2.33 ± 0.37	**0.011**	0.324	**0.011**	0.135
Dissimilarity	2.78 ± 1.60	5.17 ± 2.05	5.96 ± 2.49	**0.004**	0.077	**0.003**	0.227
SRE	0.93 ± 0.03	0.96 ± 0.06	0.96 ± 0.04	0.189	NA	NA	NA
LRE	1.29 ± 0.23	1.17 ± 0.53	1.16 ± 0.46	0.257	NA	NA	NA
LGRE	0.0052 ± 0.0060	0.0017 ± 0.0136	0.0009 ± 0.0118	**<0.001**	**0.037**	**<0.001**	**0.047**
HGRE	221 ± 253	724 ± 725	1340 ± 823	**<0.001**	**0.034**	**<0.001**	0.057
SRLGE	0.0047 ± 0.0055	0.0016 ± 0.0101	0.0009 ± 0.0095	**<0.001**	**0.040**	**<0.001**	**0.042**
SRHGE	200 ± 247	706 ± 656	1308 ± 735	**<0.001**	**0.033**	**<0.001**	0.062
LRLGE	0.0079 ± 0.0093	0.0019 ± 0.0495	0.0010 ± 0.0283	**<0.001**	**0.037**	**<0.001**	0.060
LRHGE	342 ± 282	876 ± 1242	1501 ± 2359	**<0.001**	**0.040**	**<0.001**	**0.047**
GLNUr	29.2 ± 102.4	16.9 ± 62.1	27.0 ± 29.9	0.537	NA	NA	NA
RLNU	160 ± 506	251 ± 541	416 ± 511	0.176	NA	NA	NA
RP	0.92 ± 0.05	0.95 ± 0.07	0.95 ± 0.07	0.248	NA	NA	NA
Coarseness	0.024 ± 0.015	0.018 ± 0.011	0.011 ± 0.012	0.138	NA	NA	NA
Contrast	0.24 ± 0.17	0.40 ± 0.39	0.46 ± 0.43	**0.034**	0.233	**0.028**	0.532
Busyness	0.42 ± 0.40	0.16 ± 2.37	0.22 ± 0.24	0.068	NA	NA	NA
SZE	0.58 ± 0.17	0.68 ± 0.17	0.71 ± 0.15	0.050	NA	NA	NA
LZE	27.1 ± 1004	7.65 ± 7617	9.00 ± 203	0.241	NA	NA	NA
LGZE	0.0052 ± 0.0058	0.0017 ± 0.011	0.0009 ± 0.014	**<0.001**	**0.032**	**<0.001**	**0.040**
HGZE	235 ± 241	763 ± 655	1316 ± 758	**<0.001**	**0.028**	**<0.001**	**0.049**
SZLGE	0.0029 ± 0.0022	0.0012 ± 0.0027	0.0007 ± 0.0022	**<0.001**	**0.018**	**<0.001**	**0.046**
SZHGE	135 ± 181	504 ± 545	928 ± 628	**<0.001**	**0.030**	**<0.001**	0.072
LZLGE	0.17 ± 6.38	0.01 ± 95.15	0.01 ± 10.16	**0.013**	0.122	**0.010**	0.433
LZHGE	6217 ± 168,085	6224 ± 117,521	8404 ± 508,730	0.513	NA	NA	NA
GLNUz	6.22 ± 12.7	7.38 ± 10.2	11.9 ± 14.2	0.228	NA	NA	NA
ZLNU	35.3 ± 56.7	53.0 ± 82.4	109.0 ± 155.8	**0.003**	0.720	**0.007**	**0.017**
ZP	0.31 ± 0.20	0.53 ± 0.20	0.51 ± 0.18	0.056	NA	NA	NA

Values in bold typeface indicate a significant difference. Data are presented as median ± standard deviation. NA, not applicable.

## Data Availability

The data analyzed in this study are available on written request from the corresponding author.

## Supplementary Materials

The following supporting information can be downloaded at: https://www.mdpi.com/article/10.3390/jcm13061625/s1. Table S1: Texture features; Table S2. Clinical characteristics according to PD-L1 expression levels.

## References

[1] Thai A.A., Solomon B.J., Sequist L.V., Gainor J.F., Heist R.S. (2021). Lung cancer. Lancet.

[2] Reck M., Rodríguez-Abreu D., Robinson A.G., Hui R., Csőszi T., Fülöp A., Gottfried M., Peled N., Tafreshi A., Cuffe S. (2016). Pembrolizumab versus chemotherapy for PD-L1-positive non-small-cell lung cancer. N. Engl. J. Med..

[3] Mok T.S.K., Wu Y.L., Kudaba I., Kowalski D.M., Cho B.C., Turna H.Z., Castro G., Srimuninnimit V., Laktionov K.K., Bondarenko I. (2019). Pembrolizumab versus chemotherapy for previously untreated, PD-L1-expressing, locally advanced or metastatic non-small-cell lung cancer (KEYNOTE-042): A randomised, open-label, controlled, phase 3 trial. Lancet.

[4] Aisner D.L., Riely G.J. (2021). Non-small cell lung cancer: Recommendations for biomarker testing and treatment. J. Natl. Compr. Cancer Netw..

[5] Sauter A.W., Schwenzer N., Divine M.R., Pichler B.J., Pfannenberg C. (2015). Image-derived biomarkers and multimodal imaging strategies for lung cancer management. Eur. J. Nucl. Med. Mol. Imaging.

[6] Jreige M., Letovanec I., Chaba K., Renaud S., Rusakiewicz S., Cristina V., Peters S., Krueger T., de Leval L., Kandalaft L.E. (2019). ^18^F-FDG PET metabolic-to-morphological volume ratio predicts PD-L1 tumour expression and response to PD-1 blockade in non-small-cell lung cancer. Eur. J. Nucl. Med. Mol. Imaging.

[7] Takada K., Toyokawa G., Okamoto T., Baba S., Kozuma Y., Matsubara T., Haratake N., Akamine T., Takamori S., Katsura M. (2017). Metabolic characteristics of programmed cell death-ligand 1-expressing lung cancer on 18 F-fluorodeoxyglucose positron emission tomography/computed tomography. Cancer Med..

[8] Zhao L., Liu J., Shi J., Wang H. (2020). Relationship between SP142 PD-L1 expression and ^18^F-FDG uptake in non-small-cell lung cancer. Contrast Media Mol. Imaging.

[9] Lopci E., Toschi L., Grizzi F., Rahal D., Olivari L., Castino G.F., Marchetti S., Cortese N., Qehajaj D., Pistillo D. (2016). Correlation of metabolic information on FDG-PET with tissue expression of immune markers in patients with non-small cell lung cancer (NSCLC) who are candidates for upfront surgery. Eur. J. Nucl. Med. Mol. Imaging.

[10] Ishimura M., Norikane T., Mitamura K., Yamamoto Y., Arai-Okuda H., Murota M., Ibuki E., Kanaji N., Nishiyama Y. (2022). Correlation of epidermal growth factor receptor mutation status and PD-L1 expression with [^18^F]FDG PET using volume-based parameters in non-small cell lung cancer. Nucl. Med. Commun..

[11] McGranahan N., Swanton C. (2017). Clonal heterogeneity and tumor evolution: Past, present, and the future. Cell.

[12] Kim B.S., Kang J., Jun S., Kim H., Pak K., Kim G.H., Heo H.J., Kim Y.H. (2020). Association between immunotherapy biomarkers and glucose metabolism from F-18 FDG PET. Eur. Rev. Med. Pharmacol. Sci..

[13] Zhang R., Hohenforst-Schmidt W., Steppert C., Sziklavari Z., Schmidkonz C., Atzinger A., Kuwert T., Klink T., Sterlacci W., Hartmann A. (2022). Standardized ^18^F-FDG PET/CT radiomic features provide information on PD-L1 expression status in treatment-naïve patients with non-small cell lung cancer. Nuklearmedizin.

[14] Li J., Ge S., Sang S., Hu C., Deng S. (2021). Evaluation of PD-L1 expression level in patients with non-small cell lung cancer by ^18^F-FDG PET/CT radiomics and clinicopathological characteristics. Front. Oncol..

[15] Zhou J., Zou S., Kuang D., Yan J., Zhao J., Zhu X. (2021). A novel approach using FDG-PET/CT-based radiomics to assess tumor immune phenotypes in patients with non-small cell lung cancer. Front. Oncol..

[16] Ishimura M., Norikane T., Mitamura K., Yamamoto Y., Manabe Y., Murao M., Murota M., Kanaji N., Nishiyama Y. (2023). FDG PET texture indices as imaging biomarkers for epidermal growth factor receptor mutation status in lung adenocarcinoma. Sci. Rep..

[17] Nioche C., Orlhac F., Boughdad S., Reuzé S., Goya-Outi J., Robert C., Pellot-Barakat C., Soussan M., Frouin F., Buvat I. (2018). LIFEx: A freeware for radiomic feature calculation in multimodality imaging to accelerate advances in the characterization of tumor heterogeneity. Cancer Res..

[18] Orlhac F., Soussan M., Maisonobe J.A., Garcia C.A., Vanderlinden B., Buvat I. (2014). Tumor texture analysis in ^18^F-FDG PET: Relationships between texture parameters, histogram indices, standardized uptake values, metabolic volumes, and total lesion glycolysis. J. Nucl. Med..

[19] Lambin P., Rios-Velazquez E., Leijenaar R., Carvalho S., van Stiphout R.G., Granton P., Zegers C.M., Gillies R., Boellard R., Dekker A. (2012). Radiomics: Extracting more information from medical images using advanced feature analysis. Eur. J. Cancer.

[20] Pyka T., Bundschuh R.A., Andratschke N., Mayer B., Specht H.M., Papp L., Zsótér N., Essler M. (2015). Textural features in pre-treatment [F18]-FDG-PET/CT are correlated with risk of local recurrence and disease-specific survival in early stage NSCLC patients receiving primary stereotactic radiation therapy. Radiat. Oncol..

[21] Erber R., Stöhr R., Herlein S., Giedl C., Rieker R.J., Fuchs F., Ficker J.H., Hartmann A., Veltrup E., Wirtz R.M. (2017). Comparison of PD-L1 mRNA expression measured with the CheckPoint Typer^®^ assay with PD-L1 protein expression assessed with immunohistochemistry in non-small cell lung cancer. Anticancer Res..

[22] Hatt M., Tixier F., Pierce L., Kinahan P.E., Le Rest C.C., Visvikis D. (2017). Characterization of PET/CT images using texture analysis: The past, the present…any future?. Eur. J. Nucl. Med. Mol. Imaging.

[23] Lee J.W., Lee S.M. (2018). Radiomics in oncological PET/CT: Clinical applications. Nucl. Med. Mol. Imaging.

[24] Zhang M., Li G., Wang Y., Wang Y., Zhao S., Haihong P., Zhao H., Wang Y. (2017). PD-L1 expression in lung cancer and its correlation with driver mutations: A meta-analysis. Sci. Rep..

[25] Bauml J., Seiwert T.Y., Pfister D.G., Worden F., Liu S.V., Gilbert J., Saba N.F., Weiss J., Wirth L., Sukari A. (2017). Pembrolizumab for platinum- and cetuximab-refractory head and neck cancer: Results from a single-arm, phase II study. J. Clin. Oncol..

[26] Ulas E.B., Hashemi S.M.S., Houda I., Kaynak A., Veltman J.D., Fransen M.F., Radonic T., Bahce I. (2023). Predictive value of combined positive score and tumor proportion score for immunotherapy response in advanced NSCLC. JTO Clin. Res. Rep..

[27] Borghaei H., Langer C.J., Paz-Ares L., Rodríguez-Abreu D., Halmos B., Garassino M.C., Houghton B., Kurata T., Cheng Y., Lin J. (2020). Pembrolizumab plus chemotherapy versus chemotherapy alone in patients with advanced non-small cell lung cancer without tumor PD-L1 expression: A pooled analysis of 3 randomized controlled trials. Cancer.

[28] Wang D., Qiu B., Liu Q., Xia L., Liu S., Zheng C., Liu H., Mo Y., Zhang X., Hu Y. (2023). Patlak-Ki derived from ultra-high sensitivity dynamic total body [^18^F]FDG PET/CT correlates with the response to induction immuno-chemotherapy in locally advanced non-small cell lung cancer patients. Eur. J. Nucl. Med. Mol. Imaging.

