# Supplementary material for: Texture Features of 18F-Fluorodeoxyglucose Positron Emission Tomography for Predicting Programmed Death-Ligand-1 Levels in Non-Small Cell Lung Cancer
Source: J Clin Med. 2024 Mar 12;13(6):1625. doi: 10.3390/jcm13061625 (PMC10971147; doi:10.3390/jcm13061625)
Supplement: Supplementary file 1 [file jcm-13-01625-s001.zip › jcm-2860158-supplementary.pdf]

Supplementary Materials

Table S1. Texture features.

Matrix	Index
Gray-Level Cooccurrence Matrix (GLCM)	Homogeneity<br>Energy<br>Contrast<br>Correlation<br>Entropy<br>Dissimilarity
Gray-Level Run Length Matrix (GLRLM)	Short-Run Emphasis (SRE)<br>Long-Run Emphasis (LRE)<br>Low Gray-Level Run Emphasis (LGRE)<br>High Gray-Level Run Emphasis (HGRE)<br>Short-Run Low Gray-Level Emphasis (SRLGE)<br>Short-Run High Gray-Level Emphasis (SRHGE)<br>Long-Run Low Gray-Level Emphasis (LRLGE)<br>Long-Run High Gray-Level Emphasis (LRHGE)<br>Gray-Level Non Uniformity for run (GLNUr)<br>Run Length Non Uniformity (RLNU)<br>Run Percentage (RP)
Neighborhood Gray-Level Different Matrix (NGLDM)	Coarseness<br>Contrast<br>Busyness
Gray-Level Zone Length Matrix (GLZLM)	Short-Zone Emphasis (SZE)<br>Long-Zone Emphasis (LZE)<br>Low Gray-Level Zone Emphasis (LGZE)<br>High Gray-Level Zone Emphasis (HGZE)<br>Short-Zone Low Gray-Level Emphasis (SZLGE)<br>Short-Zone High Gray-Level Emphasis (SZHGE)<br>Long-Zone Low Gray-Level Emphasis (LZLGE)<br>Long-Zone High Gray-Level Emphasis (LZHGE)<br>Gray-Level Non Uniformity for zone (GLNUz)<br>Zone Length Non Uniformity (ZLNU)<br>Zone Percentage (ZP)

Table S2. Clinical characteristics according to PD-L1 expression levels.

Characteristic		Negative PD-L1 (n = 12)	Low PD-L1 (n = 45)	High PD-L1 (n = 26)
Age (years)	Mean	77	73	75
	Range	60–92	42–87	54–85
Sex	Male	4	32	17
	Female	8	13	9
Histological subtype	Adeno	11	36	19
	Sq	1	8	6
	Adenosq	0	1	1
Clinical stage	I	3	6	2
	II	1	4	1
	III	2	6	6
	IV	6	29	17
Smoking history	Smoking	4	30	18
	Non-smoking	8	15	8

Abbreviations: Adeno, adenocarcinoma; Sq, squamous cell carcinoma; Adenosq, adenosquamous cell carcinoma; PD-L1, programmed death-ligand-1
